# Eco‐Friendly Approaches to Low‐Calorie Jam Formulation Using Tomato Waste: An Innovative and Sustainable Strategy

**DOI:** 10.1002/fsn3.70477

**Published:** 2025-08-05

**Authors:** Muhammad Hammad, Ali Imran, Muhammad Afzaal, Amar Shankar, G. V. Sivaprasad, Qaswaa Yousif Jameel, Faiyaz Ahmed, Musarrat Rasheed, Usman Naeem, Mohd Asif Shah

**Affiliations:** ^1^ Department of Food Science Government College University Faisalabad Pakistan; ^2^ Department of Food Technology, School of Engineering and Technology JAIN (Deemed to Be University) Bangalore Karnataka India; ^3^ Department of Basic Science & Humanities Raghu Engineering College Visakhapatnam India; ^4^ Department of Food Science, Colleges of Agricultural and Forestry University of Mosul Mosul Iraq; ^5^ Department of Basic Health Sciences, College of Applied Medical Sciences Qassim University Buraydah Saudi Arabia; ^6^ Department of Food Science and Technology NUR International University Lahore Pakistan; ^7^ Department of Nutritional Sciences University of Lahore Lahore Pakistan; ^8^ Department of Economics Kardan University Kabul Afghanistan; ^9^ Division of Research and Development Lovely Professional University Phagwara Punjab India; ^10^ University Centre for Research & Development, University School of Business, Chandigarh University Gharuan, Mohali Punjab India

**Keywords:** low‐caloric jam, tomato peel, tomato pomace

## Abstract

This study developed a low‐caloric jam using tomato peels and pomace and characterized their antioxidant properties. Extracts were obtained using water, ethanol, and methanol solvents at 55°C for 30, 60, and 90 min. Antioxidant analysis revealed that ethanol was the most effective solvent, followed by methanol and water. Tomato peel showed higher antioxidant activity than tomato pomace. FTIR analysis of tomato pomace extract showed C—H stretching bonds. Jam formulations were prepared using tomato peel and pomace powders, and physicochemical analysis revealed significant variations in color, texture, pH, etc., due to treatments (*p* ≤ 0.05). FTIR results showed weak peaks at 2924 cm^−1^, indicating C—H stretching bonds, which were slightly shifted to lower wave numbers, confirming the presence of bioactive compounds in the jam formulations.

## Graphical abstract

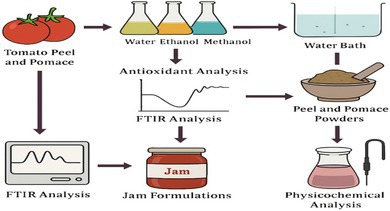

## 1 Introduction

Food waste, on the other hand, refers to a decrease in food quantity and quality as a result of decisions and actions made by customers, retailers, and other food service providers. Food loss refers to a decrease in the amount and quality of food brought on by the choices and actions of suppliers in the food chain (FAO 2019). Rapid population expansion and consumption habits are the main causes of food loss and waste (Balakrishnan et al. 2025; Kannah et al. 2020). The environmental implications of food production are made worse by food loss (Ng et al. 2020). Around 1.3 billion tons of food are produced each year for human use; roughly one‐third of that amount is lost or wasted globally. Approximately 931 million tons of food waste were produced in 2019 with homes accounting for 61% of the trash, food service providers for 26%, and merchants for 13%. Consumer purchasing power, which is frequently correlated with income levels across nations, has an impact on the output of food waste, notably from households (Dhiman et al. 2025; Ng et al. 2020). With 200 kg and 180 kg annually, respectively, Europe and Latin America have the largest consumption waste per capita rates. Oceania and North America, Central and West Asia, North Africa, industrialized Asia, sub‐Saharan Africa, and south and Southeast Asia are other areas with high consumer waste. In terms of the entire supply chain, Central and Southern Asia, Northern America, and Europe were identified as the regions with the highest food loss in 2016, encompassing post‐harvest to distribution stages (FAO 2019). High‐income countries generate approximately 307 g of food waste per capita per day, twice the amount compared to upper‐middle‐income countries (Szabo et al. 2025; Ng et al. 2020). Food waste in industrialized countries primarily occurs at later stages of the supply chain, while in developing countries, it mainly occurs at earlier stages due to inadequate financial and technical resources for harvesting, storage, and cooling (Rao and Rathod 2019). According to Obi et al. (2016), agricultural waste is made up of leftovers from unprocessed agricultural goods, such as waste from animals, agricultural waste, toxic and hazardous agricultural waste (such herbicides and pesticides), and waste from food processing in the form of liquid, slurry, or solid waste, vegetables and fruits, Cereals and pulses, meat and animal products, oil‐bearing crops tubers and roots are the key food types that contribute to nutritional and food waste or loss (Chen et al. 2020; Rao and Rathod 2019). Food waste occurs along the food chain, with homes accounting for 42%, food processing for 38%, and other operations for 20%, excluding agricultural food losses. By looking at the food distribution system, the beverage industry is responsible for 26% of food waste, which is subsequently followed by the dairy products and ice cream industry (21.3%), the production and preservation of fruit and vegetables (14.8%), the manufacturing of grain and starch products (12.9%), the production and processing of meat (8%), the production of vegetable and animal oils and fats (3.9%), the production of fish and fish products (0.4%), and the manufacturing of other food products (12.7%). The objective of the study was to develop the low‐caloric jam, and invitro characterization. Furthermore, probing physiochemical and sensorial elucidation of low‐caloric jam.

## 2 Material and Methodology

The current study was carried out in the Nutritional Lab, Department of Food Science, Government College University, Faisalabad. The aim of the current study is to develop low‐caloric jam from tomato waste (pomace and peel) and its in vitro characterization. The study comprised two experiments, that is, tomato pomace and peel extraction and functional product development.

### 2.1 Procurement of Tomato

Tomatoes were collected from the local market of Faisalabad.

### 2.2 Preparation of Tomato Peel and Pomace Powder

#### 2.2.1 Peeling

Tomatoes were washed thoroughly to remove the dust and other unwanted particles. The washed tomatoes were brought to hand peeling to separate the peel and fruit.

#### 2.2.2 Dehydration

Tomatoes were washed to remove dust and decrease microbial load with tap water was done by using the method (Al Maiman et al. 2021).

#### 2.2.3 Grinding

Dehydrated tomato peel and fruit were brought into fine powder by using a grinder.

#### 2.2.4 Proximate Analysis of Tomato Pomace and Peel Powder

Tomato pomace and peel powder were tested for water, ash, crude protein, unrefined fat, fiber, and nitrogen‐free extract (NFE) as defined by the (Committee 2000) standard. The following is a brief description of each process:

#### 2.2.5 Moisture

Place the China dish in an oven and heat it at a temperature of 135°C ± 3°C for 20 min by using (Committee 2000).

#### 2.2.6 Ash Determination

Firstly, activate the muffle furnace and allow it to heat up until it reaches a temperature of 550°C by using (Committee 2000).

#### 2.2.7 Crude Fat

The flask and thimble were placed in an oven and subjected to a temperature of 100°C for 30 min by using (Committee 2000).

#### 2.2.8 Crude Fiber

Take a 2 g portion of the previously dried and fat‐free sample, labeled as W1, and transfer it to a digestion beaker by using (Committee 2000).

#### 2.2.9 Crude Protein

Using AACC (2000) Method No. 46‐13, the Kjeldahl Apparatus (Model: D‐40599, Behr Labor Technik, Gmbh‐Germany) was employed to calculate the nitrogen content in the sample.

#### 2.2.10 Nitrogen‐Free Extracts (NFE)

The nitrogen‐free extract assay is a method used to determine the carbohydrate content in a sample by using AACC (2000).

#### 2.2.11 Conventional Solvent Extraction

Conventional extraction refers to the process of extracting valuable chemicals from plant materials using common solvents, with or without the application of heat. In the case of tomato peel and fruit extraction, the procedure described by Ali et al. (2021) was followed with some modifications.

## 3 Analysis of Extracts

### 3.1 DPPH Scavenging Activity Assay

The spectrophotometric method was employed to measure the scavenging activity of DPPH (1, 1‐diphenyl, 2‐picrylhydrazyl) as described by Roghelia and Panchal (2016).

### 3.2 ABTS (2, 2′‐Azino‐Bis, 3‐Ethylbenzothiazoline‐6‐Sulfonic Acid) Assay

The ABTS assay was conducted to estimate the activity of the g extract, following the method described by Ali et al. (2021).

### 3.3 FRAP (Ferric Reducing Antioxidant Power) Assay

In accordance with the guidelines outlined by Ali et al. (2021), the FRAP (ferric reducing antioxidant power) assay was conducted.

### 3.4 Determination of Total Flavonoid Content (TFC)

Flavonoids are the largest group of polyphenols and are known for their medicinal properties. The final absorbance was measured at 430 nm, following a modified version of the method described by (Hussain et al. 2019).

### 3.5 Determination of β‐Carotene

The extraction of beta‐carotene from tomato peel and pomace powder was performed according to Belviranlı et al. (2019), with some modifications.

### 3.6 Determination of Total Phenolic Content (TPC)

The total amount of phenolic compounds in the extracts was determined using a spectrophotometric method called the Folin‐Ciocalteau procedure, as described by Khoo et al. (2012).

## 4 Structural Analysis

### 4.1 Fourier Transform Infrared Spectroscopy (FTIR)

FTIR measurements were conducted using a Bruker model IFS 32 spectrometer. FTIR of tomato peel and tomato pomace powder was performed in the Department of Chemistry, University of Agriculture, Faisalabad. Spectra were generated in the spectral region of 4000 to 650 cm^−1^ by averaging 40 scans (Valenzuela et al. 2013).

### 4.2 Product Development

**FIGURE 1:**
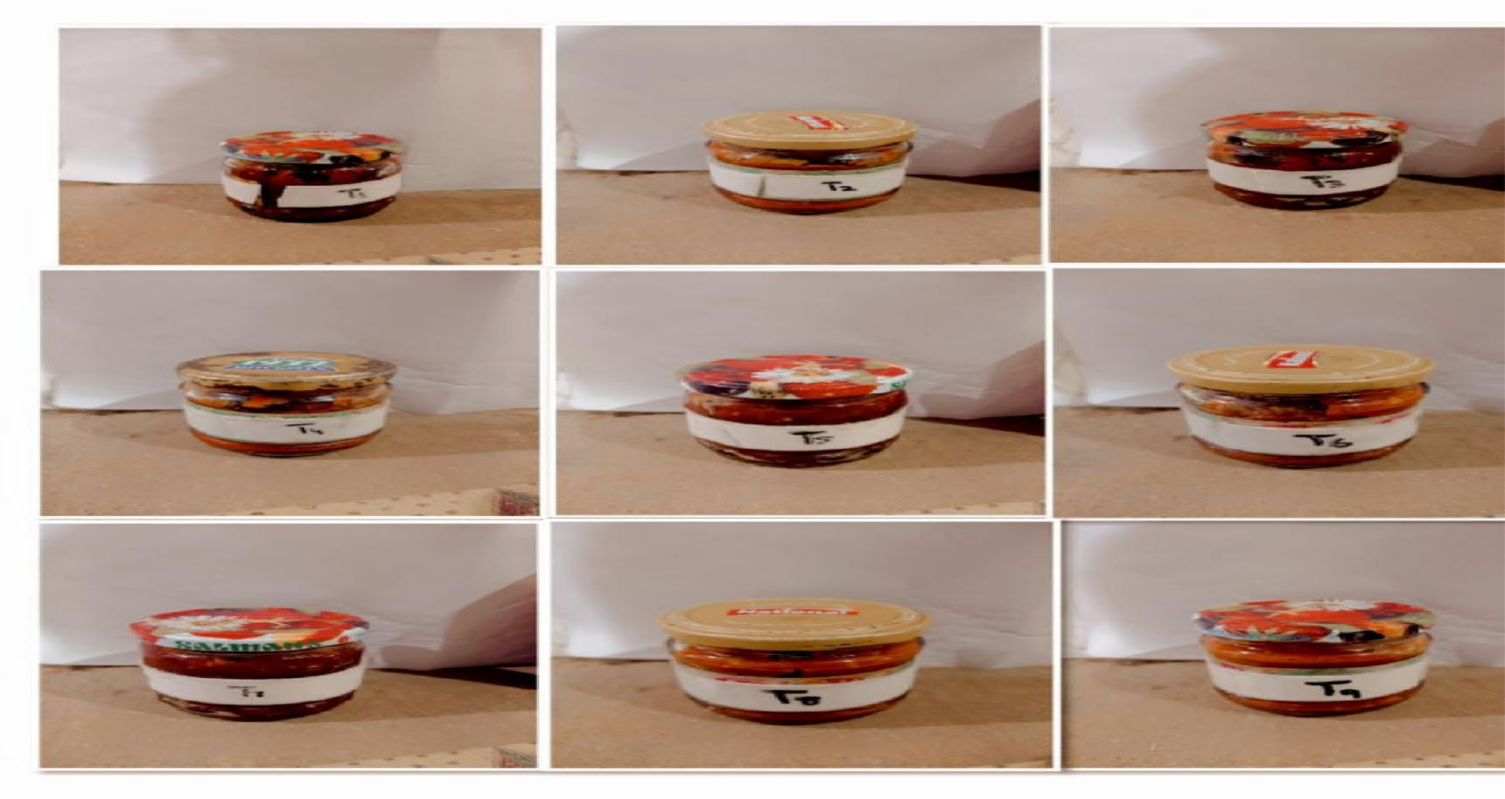
Jam formulations. T_1_: Jam with tomato peel powder (15%), T_2_: Jam with tomato peel powder (17%), T_3_: Jam with tomato peel powder (19%), T_4_: Jam with tomato pomace powder (15%), T_5_: Jam with tomato pomace powder (17%), T_6_: Jam with tomato pomace powder (19%), T_7_: Jam with tomato peel and powder (7.5% + 7.5%), T_8_: Jam with tomato peel and powder (8.5% + 8.5%), T_9_: Jam with tomato peel and powder (9.5% + 9.5%).

In the product development phase, ten different treatments of functional tomato jam were prepared in a completely hygienic environment. Each treatment was labeled accordingly. The control sample, labeled as T0, was included for comparison purposes. T_1_ sample was prepared using 15% tomato peel powder, T_2_ sample was prepared using 17% tomato peel powder, and T_3_ sample was prepared using 19% tomato peel powder. T_4_ was prepared using 15% tomato pomace powder, T_5_ was prepared using 17% tomato pomace powder, and T_6_ was prepared using 19% tomato pomace powder. T_7_ was a combination of 7.5% tomato pomace powder and 7.5% peel powder, T_8_ was a combination of 8.5% tomato pomace powder and 8.5% peel powder, and T_9_ was a combination of 9.5% tomato pomace powder and 9.5% peel powder in Figure 1.

## 5 Physiochemical Analysis

### 5.1 Color

The color of the prepared jam was determined using a Hunter color lab meter, following the method described by (Serna‐Saldivar 2012).

### 5.2 Texture

It is important to note that the specific procedures and parameters may vary depending on the type of sample being analyzed and the desired texture attributes. Standardized methods, such as those provided by organizations like the American Society of Testing and Materials (ASTM) or the International Organization for Standardization (ISO), can be followed for specific applications or industries.

### 5.3 pH

pH of the jam was determined by using a digital pH meter (Eutech pH 700 Meter) by following (Serna‐Saldivar 2012).

### 5.4 Sensory Evaluation

Sensory analysis offers a valuable opportunity to comprehensively evaluate various properties of a product based on human perception through the five senses. It serves as an important tool for assessing the quality and control of newly developed products. In this study, a sample of jam was presented to a panel of 10 sensory evaluation experts. The evaluation focused on attributes such as appearance, color, flavor, taste, aroma, and overall consumer acceptability. The evaluations were conducted at intervals of 0, 7, 14, and 28 days. The panelists were provided with a detailed method and score sheet, employing a 9‐point hedonic scale, to guide their assessments. The average values were analyzed, and higher scores indicated better quality attributes (with a scale ranging from 1 for extremely disliked to 9 for extremely liked) (Saeed et al. 2021). The hedonic responses were assessed in the Sensory Evaluation Laboratory of the Department of Food Sciences at Government College University, Faisalabad.

### 5.5 Statistical Analysis

Analysis of variance with triplicates, mean, and standard deviation with a 95% confidence interval was carried out to determine the difference between the mean, and significance level was observed at *p* ≤ 0.05 by using Statistix 8.1 (Roghelia and Panchal 2016).

### 5.6 Proximate Composition of Tomato Peel and Pomace

Proximate analysis of any product is important and is used for assessing the quality of the product or any raw material. Tomato peel and pomace powder subjected to different quality checking traits and revealed moisture, ash, fat, fiber, protein, and NFE in peel were 5.70 ± 0.91, 3.01 ± 0.85, 6.01 ± 0.1, 60.5 ± 2.58, 13.05 ± 0.97, and 19.46% ± 0.95% and in pomace 9.25 ± 0.72, 4.23 ± 0.66, 1.88 ± 0.04, 45.38 ± 1.85, 18.45 ± 1.5, and 30.06% ± 2.32%, respectively, Table 1.

**TABLE 1:** Total phenolic content of tomato peel and pomace powder.

Peel					Pomace					Peel + Pomace					*F*	
Solvent	Time				Solvent	Time				Solvent	Time					
	**30**	**60**	**90**	**Mean**		**30**	**60**	**90**	**Mean**		**30**	**60**	**90**	**Mean**	**Solvent**	2.43**
Water	0.57 ± 0.04	0.73 ± 0.06	0.64 ± 0.05	0.62 ± 0.05c	Water	0.54 ± 0.05	0.71 ± 0.06	0.61 ± 0.05	**0.62 ± 0.06c**	Water	1.162 ± 0.1	1.17 ± 0.09	1.17 ± 0.09	**1.17 ± 0.10a**	Treatment	4.76**
Ethanol	0.62 ± 0.05	0.63 ± 0.06	0.70 ± 0.06	0.72 ± 0.06a	Ethanol	0.58 ± 0.05	0.75 ± 0.06	0.65 ± 0.05	**0.66 ± 0.06a**	Ethanol	0.61 ± 0.05	0.69 ± 0.05	0.79 ± 0.07	**0.70 ± 0.09b**	Time	7.01*
Methanol	0.60 ± 0.05	0.77 ± 0.06	0.67 ± 0.05	0.69 ± 0.05b	Methanol	0.56 ± 0.05	0.72 ± 0.06	0.63 ± 0.05	**0.63 ± 0.06b**	Methanol	0.59 ± 0.05	0.66 ± 0.06	0.76 ± 0.07	**0.67 ± 0.06b**	Interaction	13.21 ^(NS)^
Mean	**0.60 ± 0.05c**	**0.78 ± 0.06a**	**0.68 ± 0.05b**		Mean	**0.77 ± 0.07**	**0.89 ± 0.08**	**0.83 ± 0.07**		Mean	**0.79 ± 0.07c**	**0.85 ± 0.08b**	**0.91 ± 0.08a**			

*Note:* Values are presented as mean ± SD (*n* = 3). Values imparted by different letters in columns and rows were significantly different (*p* ≤ 0.05). Bold values highlight the highest or most significant mean values in each comparison.

* Indicates significance at *p* ≤ 0.05.

** Indicates significance at *p* ≤ 0.01.

The findings of the current investigation on the proximate composition of tomato peel powder align with the observations made by Navarro‐González et al. (2011). Their study reported the following percentages: moisture (5.71%), ash (3%), lipid (6%), total dietary fiber (TDF) (62.16 g/100 g), and crude protein (13.3%). Similarly, Al‐Qahtani et al. (2020) provided details about the nutritional composition of tomato pomace powder, which included the following percentages: moisture (80.16%), ash (12.61%), protein (3.45%), fat (2.14%), fiber (32%), and total carbohydrates (72%). In a previous study, Fuentes et al. (2013) evaluated the composition of tomato pomace and found the following percentages: protein (16%), ash (4%), fiber (46%), and fat (2%). Subsequently, Wang et al. (2016) conducted research on dried tomato peel and observed the proximate composition to be as follows: fat (4.94%–6%), protein (11.02%–11.13%), ash (4.92%–6.46%), and fiber (74.46%–76.67%).

## 6 Antioxidant Analysis of Extracts

The antioxidant activity of extracts was determined by TPC, DPPH, TFC, FRAP, ABTS, and β‐carotene.

### 6.1 TPC of Tomato Peel and Pomace Powder

Polyphenols, a group of substances known as free radical terminators, are responsible for the reduction of metal oxides, resulting in a blue color and a broad range of light absorption with a peak at 765 nm.

The F value presented in (Table 1) revealed a significant effect of treatment, solvent, and time on the TPC of tomato peel and pomace extracts. However, their interactive effect did not yield significant variations. The mean values for TPC indicated that tomato pomace had a higher content compared to tomato peel. Among the solvents, ethanol performed better in terms of extraction compared to methanol and water. Additionally, the highest extraction was observed at 90 min, followed by 60 min and 30 min.

The highest TPC content in tomato pomace was observed in ethanol (0.66 ± 0.06 mg GAE/g), followed by methanol (0.63 ± 0.06 mg GAE/g), and water (0.62 ± 0.06 mg GAE/g). Similarly, in tomato peel, the highest TPC content was observed in ethanol (0.72 ± 0.062 mg GAE/g), followed by methanol (0.69 ± 0.05 mg GAE/g), and the lowest in water (0.62 ± 0.05 mg GAE/g). Regarding the variation in time, the maximum TPC content for peel was 0.78 ± 0.064 mg GAE/g at 60 min, followed by 90 min (0.68 ± 0.058 mg GAE/g), and the minimum at the initial time of 30 min (0.60 ± 0.052 mg GAE/g). Furthermore, for pomace, the TPC values were 0.89 ± 0.08 mg GAE/g, 0.83 ± 0.075 mg GAE/g, and 0.77 ± 0.07 mg GAE/g at 60 min, 90 min, and 30 min, respectively, as mentioned in Table 1.

According to Fuentes et al. (2013), the phenolic contents exhibited a decreasing order as follows: peels (36.9 ± 0.8) > pulp (33.3 ± 0.5) > seeds (17.6 ± 0.9) mg GAE/100 g, respectively. The differences observed among the tested samples were statistically significant (*p* < 0.05). In a separate study by (Jamaleddine et al. 2022), the TPC was measured for the extracts. The highest TPC values were obtained for extracts in ethanol, which measured 99.8 mg GAE/g extract. Similarly, the ethanol: water mixture yielded a TPC of 93.0 mg GAE/g extract.

### 6.2 DPPH Scavenging of Tomato Peel and Pomace Powder

The DPPH assay, which measures the ability of samples to scavenge the stable nitrogen radical DPPH, is commonly used to evaluate free radical scavenging activity. There is a positive correlation between the extract's ability to scavenge DPPH radicals and a decrease in color. The mean values (Table 2) for DPPH extraction indicated a significant effect of treatment, time, and solvent. However, their interactive effect did not yield significant differences. The mean values for DPPH activity showed that peel exhibited the highest values compared to pomace. Similarly, among the time intervals, the 60‐min interval showed better extraction than 90 and 30 min. Additionally, ethanol exhibited the highest DPPH activity, followed by methanol and water.

**TABLE 2:** DPPH scavenging of tomato peel and pomace powder.

Peel					Pomace					Peel + Pomace					*F*	
Solvent	Time				Solvent	Time				Solvent	Time					
	**30**	**60**	**90**	**Mean**		**30**	**60**	**90**	**Mean**		**30**	**60**	**90**	**Mean**	**Solvent**	3.84**
Water	22.45 ± 1.71	24.55 ± 1.94	23.35 ± 1.84	**23.43 ± 2.15c**	Water	22.00 ± 1.74	23.43 ± 1.85	21.87 ± 1.65	**22.43 ± 1.81b**	Water	23.54 ± 1.85	23.12 ± 1.87	22.32 ± 1.62	**22.99 ± 1.74c**	Treatment	5.76**
Ethanol	24.69 ± 1.94	27.00 ± 2.23	25.68 ± 1.97	**25.80 ± 2.51a**	Ethanol	22.96 ± 1.79	25.11 ± 2.01	23.88 ± 1.86	**23.00 ± 1.98a**	Ethanol	26.62 ± 1.56	25.32 ± 1.98	24.34 ± 1.91	**25.42 ± 2.01a**	Time	8.32*
Methanol	23.68 ± 1.82	25.90 ± 1.98	24.63 ± 1.93	**24.74 ± 1.96b**	Methanol	22.90 ± 1.76	24.08 ± 1.91	22.90 ± 1.78	**23.98 ± 1.81a**	Methanol	25.53 ± 1.97	24.28 ± 1.93	23.34 ± 1.87	**24.38 ± 1.91b**	Interaction	12.45 ^(NS)^
Mean	**23.61 ± 2.03c**	**25.82 ± 2.36a**	**24.56 ± 2.12b**		Mean	**22.33 ± 2.16b**	**24.21 ± 2.38a**	**22.88 ± 2.13b**		Mean	**26.42 ± 2.01a**	**24.18 ± 1.92b**	**23.00 ± 1.87c**			

*Note:* Values are presented as mean ± S.D (*n* = 3). Values imparted by different letters in columns and rows were significantly different (*p* ≤ 0.05). Bold values highlight the highest or most significant mean values in each comparison.

* Indicates significance at *p* ≤ 0.05.

** Indicates significance at *p* ≤ 0.01.

The highest DPPH value in tomato peel was observed at 60 min (25.82% ± 2.36%), followed by 90 min (24.56% ± 2.12%), and 30 min (23.61% ± 2.03%). Similar trends were observed in tomato pomace, with DPPH values at 30, 60, and 90 min recorded as 22.332% ± 2.16%, 24.210% ± 2.38%, and 22.888% ± 2.13%, respectively. Among the solvents, ethanol exhibited the highest DPPH values, with 25.80% ± 2.51% for peel, followed by methanol (24.74% ± 1.96%), and the lowest value was observed in water (23.43% ± 2.15%). Similarly, the recorded values of DPPH for pomace were 23.98% ± 1.81% in ethanol, followed by 23.00% ± 1.98% in methanol, and 22.435% ± 1.81% in water, as presented in (Table 2).

The results of this study align with the findings reported by Vorobyova et al. (2022), who examined the DPPH content of tomato peel using ethanol at various concentrations, ranging from 29.06% ± 0.91% to 31.725% ± 1.23%. The minor variations in outcomes could be attributed to the utilization of a different tomato variety in their research. In another study conducted by Jamaleddine et al. (2022), the DPPH content of tomato pomace extract was assessed as a percentage inhibition. The research concluded that the tomato pomace extract exhibited radical scavenging activity, with the highest antioxidant activities (69.7% and 71.6% inhibition) observed in extracts obtained using ethanol:water and ethanol.

### 6.3 Flavonoid Content of Tomato Peel and Pomace Powder

The F value indicated a significant effect of treatment, time, and solvent on the flavonoid content of tomato peel and pomace extract. However, their interactive effect did not yield significant results, as shown in Table 3. The mean values for the effect of time and solvent intervals on flavonoid content demonstrated that tomato peel had higher values compared to pomace. Similarly, among the time intervals, the 60‐min interval showed better extraction than 90 and 30 min. Additionally, ethanol exhibited the highest mean flavonoid content, followed by methanol and water.

**TABLE 3:** Flavonoid content of tomato peel and pomace powder.

Peel					Pomace					Peel + Pomace					*F*	
Solvent	Time				Solvent	Time				Solvent	Time					
	**30**	**60**	**90**	**Mean**		**30**	**60**	**90**	**Mean**		**30**	**60**	**90**	**Mean**	**Solvent**	3.54**
Water	0.014 ± 0.001	0.015 ± 0.001	0.014 ± 0.001	**0.014 ± 0.001c**	Water	0.010 ± 0.001	0.012 ± 0.001	0.011 ± 0.0001	**0.011 ± 0.001c**	Water	0.012 ± 0.001	0.014 ± 0.001	0.012 ± 0.001	**0.012 ± 0.001c**	Treatments	5.24**
Ethanol	0.015 ± 0.001	0.017 ± 0.001	0.016 ± 0.001	**0.016 ± 0.001a**	Ethanol	0.014 ± 0.001	0.015 ± 0.001	0.015 ± 0.001	**0.015 ± 0.0012a**	Ethanol	0.015 ± 0.001	0.016 ± 0.0001	0.015 ± 0.001	**0.016 ± 0.001a**	Time	9.45*
Methanol	0.015 ± 0.001	0.016458 ± 0.001	0.015 ± 0.001	**0.015 ± 0.001b**	Methanol	0.013 ± 0.001	0.015 ± 0.001	0.014 ± 0.001	**0.014 ± 0.001b**	Methanol	0.014 ± 0.001	0.016 ± 0.001	0.015 ± 0.001	**0.015 ± 0.001b**	Interaction	11.87 ^(NS)^
Mean	**0.014 ± 0.001c**	**0.016 ± 0.001a**	**0.015 ± 0.001b**		Mean	**0.012 ± 0.009b**	**0.014 ± 0.0019a**	**0.014 ± 0.001a**			**0.013 ± 0.001b**	**0.014 ± 0.001a**	**0.014 ± 0.001a**			

*Note:* Values are presented as mean ± SD (*n* = 3). Values imparted by different letters in columns and rows were significantly different (*p* ≤ 0.05). Bold values highlight the highest or most significant mean values in each comparison.

* Indicates significance at *p* ≤ 0.05.

** Indicates significance at *p* ≤ 0.01.

The highest flavonoid content in tomato peel was observed at 0.0016% ± 0.0001% after 60 min, followed by 0.0156% ± 0.0012% and 0.0149% ± 0.001% at 90 and 30 min, respectively. Moreover, in tomato pomace, the flavonoid content was observed as 0.0145% ± 0.0019%, 0.0140% ± 0.0017%, and 0.0129% ± 0.0009% at 60, 90, and 30 min, respectively. Among the solvents, ethanol exhibited the highest flavonoid content, with values of 0.0164% ± 0.001% for peel and 0.0154% ± 0.0012% for pomace. This was followed by methanol with values of 0.0157% ± 0.001% for peel and 0.0146% ± 0.001% for pomace. The lowest flavonoid content was observed in water, with values of 0.0149% ± 0.0001% for peel and 0.0113% ± 0.001% for pomace. These values are presented in Tables 3, 4.

**TABLE 4:** ABTS of tomato peel and pomace powder.

Peel					Pomace					Peel + Pomace					*F*	
Solvent	Time				Solvent	Time				Solvent	Time					
	**30**	**60**	**90**	**Mean**		**30**	**60**	**90**	**Mean**		**30**	**60**	**90**	**Mean**	**Solvent**	2.43**
Water	121.87 ± 9.76	127.8 ± 10.27	122.54 ± 9.97	**124.07 ± 9.98c**	Water	87.21 ± 6.97	97.55 ± 7.23	98.33 ± 7.87	**94.36 ± 6.96c**	Water	112.6 ± 8.75	119.8 ± 9.43	113.9 ± 8.87	**115.43 ± 9.04c**	Treatments	5.73**
Ethanol	134.05 ± 11.02	140.58 ± 12.67	134.79 ± 11.74	**136.48 ± 11.53a**	Ethanol	124.67 ± 9.98	130.73 ± 11.23	125.35 ± 10.02	**126.92 ± 10.12a**	Ethanol	132.15 ± 10.76	138.58 ± 11.35	132.87 ± 10.73	**134.53 ± 10.97a**	Time	9.37*
Methanol	128.57 ± 10.43	134.82 ± 11.54	129.27 ± 11.01	**130.89 ± 10.98b**	Methanol	119.57 ± 8.76	125.39 ± 10.35	120.23 ± 9.56	**121.73 ± 9.68b**	Methanol	126.74 ± 10.13	132.91 ± 10.78	127.44 ± 10.19	**129.03 ± 1.45b**	Interactions	12.84 ^(NS)^
Mean	**128.17 ± 10.35b**	**134.40 ± 11.56a**	**128.87 ± 10.87b**		Mean	**110.48 ± 7.57c**	**117.89 ± 9.56a**	**114.63 ± 8.97b**		Mean	**123.83 ± 9.89c**	**130.43 ± 10.54a**	**124.73 ± 10.92b**			

*Note:* Values are presented as mean ± S.D (*n* = 3). Values imparted by different letters in columns and rows were significantly different (*p* ≤ 0.05). Bold values highlight the highest or most significant mean values in each comparison.

* Indicates significance at *p* ≤ 0.05.

** Indicates significance at *p* ≤ 0.01.

According to Nour et al. (2018) research, they discovered that tomato pomace contained 0.4 mg of RE/g when extracted using methanol with sonication. However, when they used ethanol, the calculated value of TFC was 3.08 mg RE/g dry weight of pomace, indicating that ethanol is a suitable solvent for extracting flavonoids from tomato pomace (Nour et al. 2018). Our results are in line with the findings of Nour et al. (2018). In another study conducted by Vorobyova et al. (2022), the TFC of tomato pomace extract was 8.74 ± 1.20 when they used ethanol. Overall, these variations may be attributed to different factors such as the source of the tomato samples, sample preparation methods, and variations in analytical techniques employed in the different studies (Vorobyova et al. 2022).

#### 6.3.1 ABTS of Tomato Peel and Pomace Powder

The F value indicated a significant effect of treatment, time, and solvent intervals on the ABTS value of tomato peel and pomace extract. The mean values for the effect of time and solvent intervals on ABTS activity demonstrated that tomato peel exhibited higher values compared to pomace. Similarly, among the time intervals, the 60‐min interval showed better extraction than 90 and 30 min. Additionally, ethanol exhibited the highest mean ABTS activity, followed by methanol and water.

The highest ABTS value in tomato peel was observed in the ethanol extract, with a value of 136.48 ± 11.53 μg/mL, followed by 130.89 ± 10.98 μg/mL in methanol and 124.07 ± 9.98 μg/mL in water. Furthermore, the ABTS activity of pomace was observed as 94.363 ± 6.96 μg/mL, 121.7313 ± 9.68 μg/mL, and 126.9236 ± 10.12 μg/mL in water, methanol, and ethanol, respectively, which were significantly different. When considering the time intervals, the highest ABTS activity for both peel and pomace was observed at 60 min, with values of 134.829 ± 11.54 μg/mL and 117.8935 ± 9.56 μg/mL, respectively. This was followed by the 90‐min interval, with values of 128.87 ± 10.87 μg/mL for peel and 114.63 ± 8.97 μg/mL for pomace. The lowest activity was observed at the initial time interval of 30 min, with values of 128.17 ± 10.35 μg/mL for peel and 110.4853 ± 7.57 μg/mL for pomace, as shown in Table 4.

#### 6.3.2 FRAP of Tomato Peel and Pomace Powder

The ferric reducing antioxidant power (FRAP) assay is a method used to determine the overall antioxidant strength, also known as reducing capacity.

The *F* value revealed that the treatment, time, and solvent had a significant effect on the FRAP activity of tomato peel and pomace extracts. Similarly, their interactive effect showed non‐significant variations, as shown in Table 5.

**TABLE 5:** FRAP of tomato peel and pomace powder.

Peel					Pomace					Peel + Pomace					*F*	
Solvent	Time				Solvent	Time				Solvent	Time					
	**30**	**60**	**90**	**Mean**		**30**	**60**	**90**	**Mean**		**30**	**60**	**90**	**Mean**	**Solvent**	3.71**
Water	17.99 ± 1.23	18.36 ± 1.23	19.63 ± 1.54	**18.66 ± 1.32c**	Water	15.67 ± 1.21	16.17 ± 1.12	17.77 ± 1.25	**16.53 ± 1.10c**	Water	16.57 ± 1.12	17.23 ± 1.25	18.12 ± 1.32	**17.30 ± 1.25c**	Treatments	5.83**
Ethanol	19.78 ± 1.45	25.01 ± 2.01	21.59 ± 1.67	**22.13 ± 1.67a**	Ethanol	18.40 ± 1.34	23.26 ± 1.87	20.08 ± 1.45	**20.58 ± 1.55a**	Ethanol	19.50 ± 1.54	24.66 ± 1.98	21.28 ± 1.64	**21.81 ± 1.67a**	Time	10.37*
Methanol	18.97 ± 1.32	19.61 ± 1.45	20.70 ± 1.51	**19.77 ± 1.45b**	Methanol	17.65 ± 1.24	18.23 ± 1.32	19.25 ± 1.44	**18.38 ± 1.32b**	Methanol	18.70 ± 1.34	19.33 ± 1.53	20.41 ± 1.54	**19.48 ± 1.45b**	Interactions	12.83 ^(NS)^
Mean	**18.92 ± 1.31c**	**21.00 ± 1.67a**	**20.64 ± 1.56b**		Mean	**17.24 ± 1.24b**	**19.22 ± 1.42a**	**19.03 ± 1.42a**		Mean	**18.26 ± 1.34c**	**20.40 ± 1.53a**	**19.94 ± 1.43b**			

*Note:* Values are presented as mean ± SD (*n* = 3). Values imparted by different letters in columns and rows were significantly different (*p* ≤ 0.05). Bold values highlight the highest or most significant mean values in each comparison.

* Indicates significance at *p* ≤ 0.05.

** Indicates significance at *p* ≤ 0.01.

The mean values presented in (Table 5) indicated that the minimum FRAP activity was observed at the initial time of 30 min for both peel and pomace extracts, with values of (18.92 ± 1.31 μmol Fe2+/g) and (17.24 ± 1.24 μmol Fe2+/g), respectively. The FRAP activity increased at 90 min to (20.64 ± 1.56 μmol Fe2+/g) for peel and (19.03 ± 1.42 μmol Fe2+/g) for pomace. The maximum FRAP activity was observed at 60 min, with values of (21.00 ± 1.67 μmol Fe2+/g) for peel and (19.22 ± 1.48 μmol Fe2+/g) for pomace.

Furthermore, among the solvent intervals, the highest FRAP activity for both peel and pomace extracts was observed in ethanol, with values of (22.13 ± 1.67 μmol Fe2+/g) and (20.58 ± 1.55 μmol Fe2+/g), respectively. This was followed by methanol, with values of (19.77 ± 1.45 μmol Fe2+/g) for peel and (18.38 ± 1.32 μmol Fe2+/g) for pomace. The lowest FRAP activity was observed in water, with values of (18.66 ± 1.32 μmol Fe2+/g) for peel and (16.53 ± 1.10 μmol Fe2+/g) for pomace.

According to Fuentes et al. (2013), among the samples tested, red tomato peels showed significantly higher FRAP values (46.9 ± 0.9 μmol Fe2+/g) compared to the pulp (31.8 ± 0.9 μmol Fe2+/g) and seeds mucilage (25.5 ± 0.8 μmol Fe2+/g) in red tomatoes, with statistically significant differences (*p* < 0.05). The slight differences in results might be attributed to the use of a different solvent and tomato variety in their study.

#### 6.3.3 β‐Carotene Content of Tomato Peel and Pulp

The *F* value of the β‐carotene content revealed a significant effect of treatment, time intervals, and solvent intervals. However, the interactive effect of these factors showed non‐significant effects, as indicated in (Table 6). The mean values of β‐carotene content demonstrated that the peel had the highest value compared to the pomace. Similarly, among the time intervals, the 60‐min interval showed the highest extraction, followed by 90 and 30 min. Regarding solvents, ethanol exhibited the highest mean β‐carotene content, followed by methanol and water.

**TABLE 6:** βeta‐carotene of tomato peel and pomace powder.

Peel					Pomace					Peel + Pomace					*F*	
Solvent	Time				Solvent	Time				Solvent	Time					
	**30**	**60**	**90**	**Mean**		**30**	**60**	**90**	**Mean**		**30**	**60**	**90**	**Mean**	**Solvent**	2.74**
Water	23.93 ± 1.87	35.26 ± 3.12	28.86 ± 2.21	**29.35 ± 2.40c**	Water	22.03 ± 1.75	32.65 ± 2.74	27.53 ± 2.34	**27.40 ± 2.71c**	Water	28.07 ± 2.31	34.98 ± 2.93	23.86 ± 1.87	**28.97 ± 2.34b**	Treatments	4.95**
Ethanol	26.33 ± 2.11	38.78 ± 3.23	31.74 ± 2.71	**32.29 ± 2.51a**	Ethanol	24.48 ± 1.93	36.07 ± 3.12	29.52 ± 2.45	**30.08 ± 7.46a**	Ethanol	31.29 ± 2.75	38.23 ± 3.21	25.95 ± 1.95	**31.83 ± 2.78a**	Time	8.57*
Methanol	25.25 ± 1.92	37.20 ± 3.21	30.44 ± 2.54	**30.97 ± 2.52b**	Methanol	23.48 ± 1.87	34.59 ± 1.92	28.31 ± 2.34	**28.80 ± 2.31b**	Methanol	30.01 ± 2.54	36.67 ± 2.91	24.89 ± 1.94	**30.52 ± 2.54ab**	Interaction	11.48 ^(NS)^
Mean	**25.17 ± 2.26c**	**37.08 ± 3.28a**	**30.35 ± 2.78b**		Mean	**23.33 ± 2.01c**	**34.43 ± 3.24a**	**28.45 ± 2.67b**		Mean	**29.84 ± 2.41b**	**36.47 ± 2.91a**	**23.17 ± 1.87c**			

*Note:* Values are presented as mean ± SD (*n* = 3). Values imparted by different letters in columns and rows were significantly different (*p* ≤ 0.05). Bold values highlight the highest or most significant mean values in each comparison.

* Indicates significance at *p* ≤ 0.05.

** Indicates significance at *p* ≤ 0.01.

The concluded results, as presented in Table 6, showed the mean β‐carotene content in the pomace at 60, 90, and 30 min to be 34.43 ± 3.24, 28.45 ± 2.67, and 23.33 ± 2.01, respectively. Likewise, the highest β‐carotene value was observed in the peel at 60 min (37.08 ± 3.28), followed by 90 min (30.35 ± 2.78), and the lowest at 30 min (25.17 ± 2.26). However, among the solvent extractions of the peel and pomace, ethanol exhibited the highest β‐carotene content, with values of (32.29 ± 2.51) and (30.08 ± 7.46), respectively. This was followed by methanol with values of (30.97 ± 2.52) and (28.80 ± 2.31), respectively. Water provided the least amount of β‐carotene from the peel (29.35 ± 2.40) and pomace (27.40 ± 2.71).

The results of this study align with the findings reported by Azabou et al. (2020), who assessed the β‐carotene bleaching inhibition activity (%) of tomato pomace extract using ethanol at different concentrations, ranging from 44.46% ± 0.12% at 100 μg/mL to 56.34% ± 0.86% at 200 μg/mL (Azabou et al. 2020). The minor variations in results can be attributed to the utilization of a different tomato variety in their research. In another investigation conducted by Jamaleddine et al. (2022), the β‐carotene content of tomato pomace extract was measured, and the study concluded that the highest antioxidant activities (82 mg/g extract and 128.2 mg/g extract) were observed for extracts obtained using various solvents.

### 6.4 Structural Analysis

#### 6.4.1 FTIR (Fourier Transform Infrared Spectroscopy)

**FIGURE 2:**
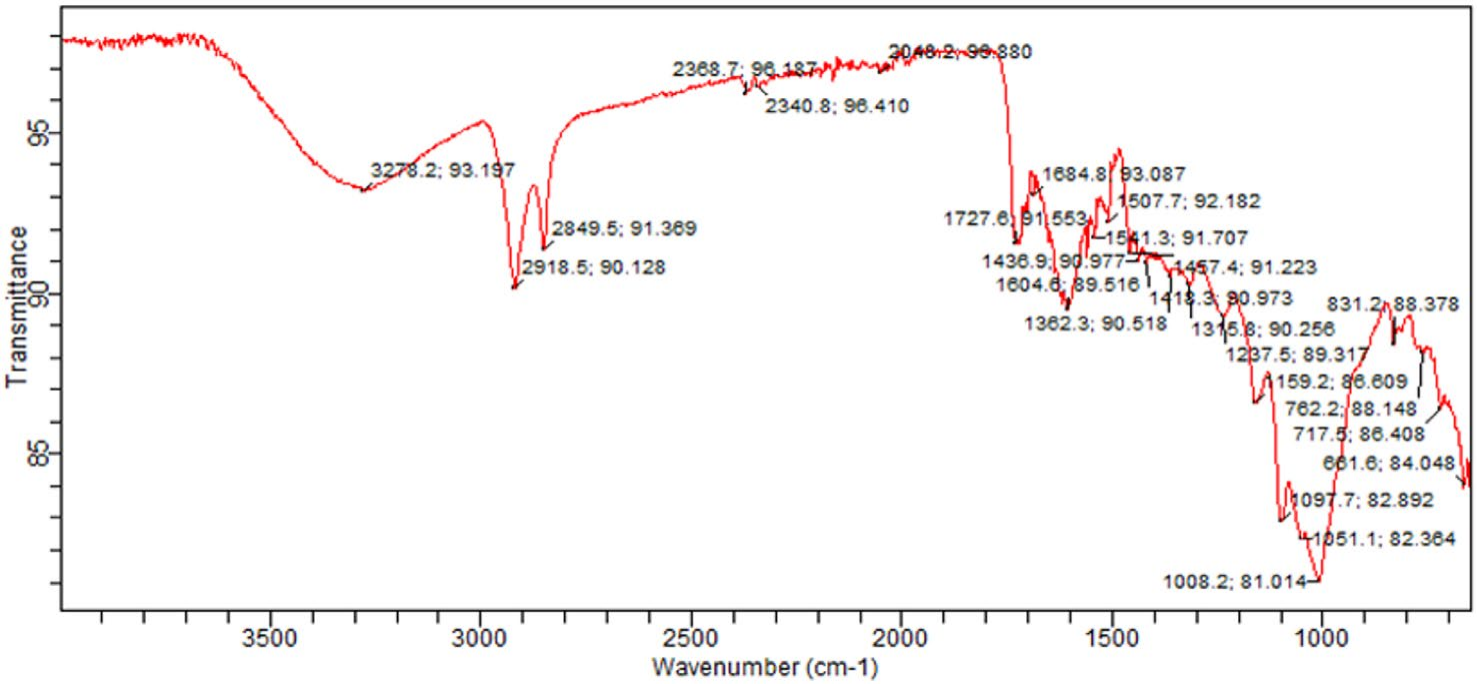
FTIR of tomato peel.

**FIGURE 3:**
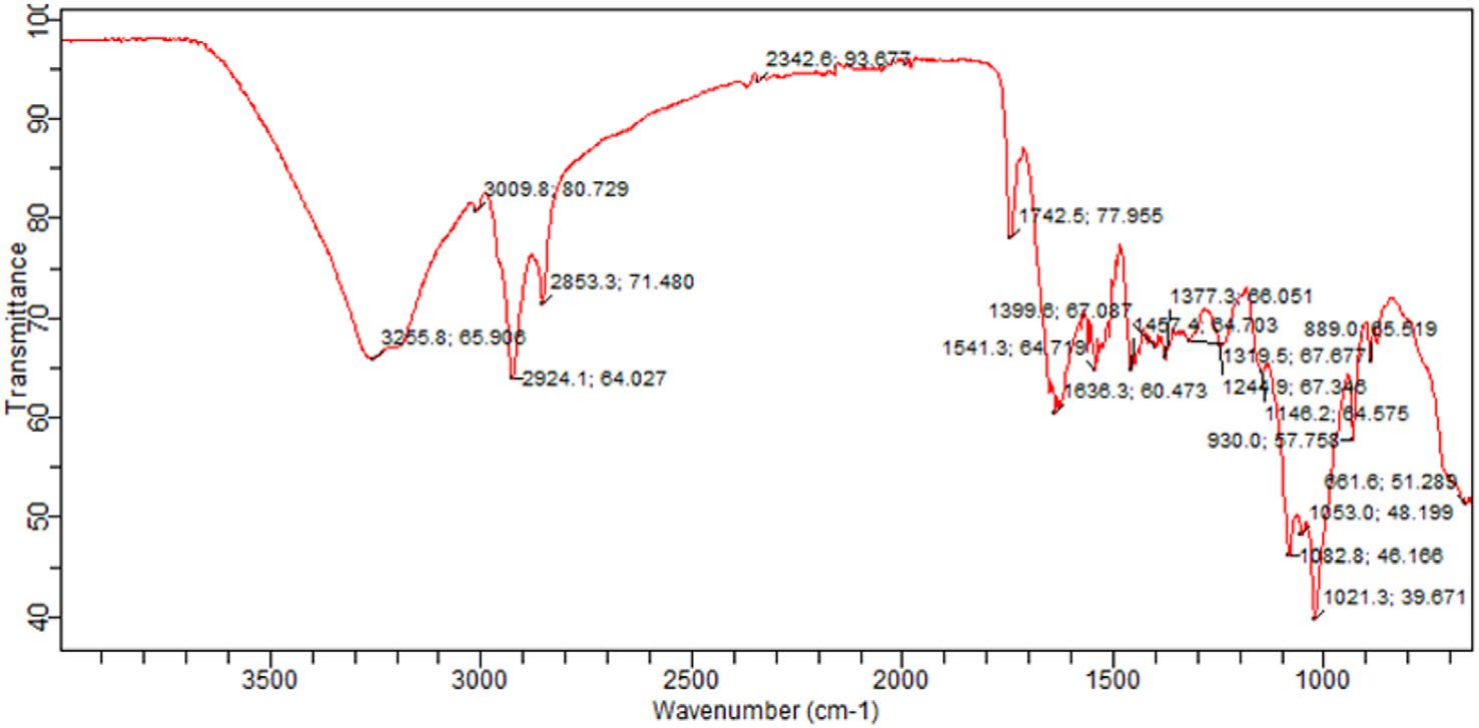
FTIR of tomato pomace.

**FIGURE 4:**
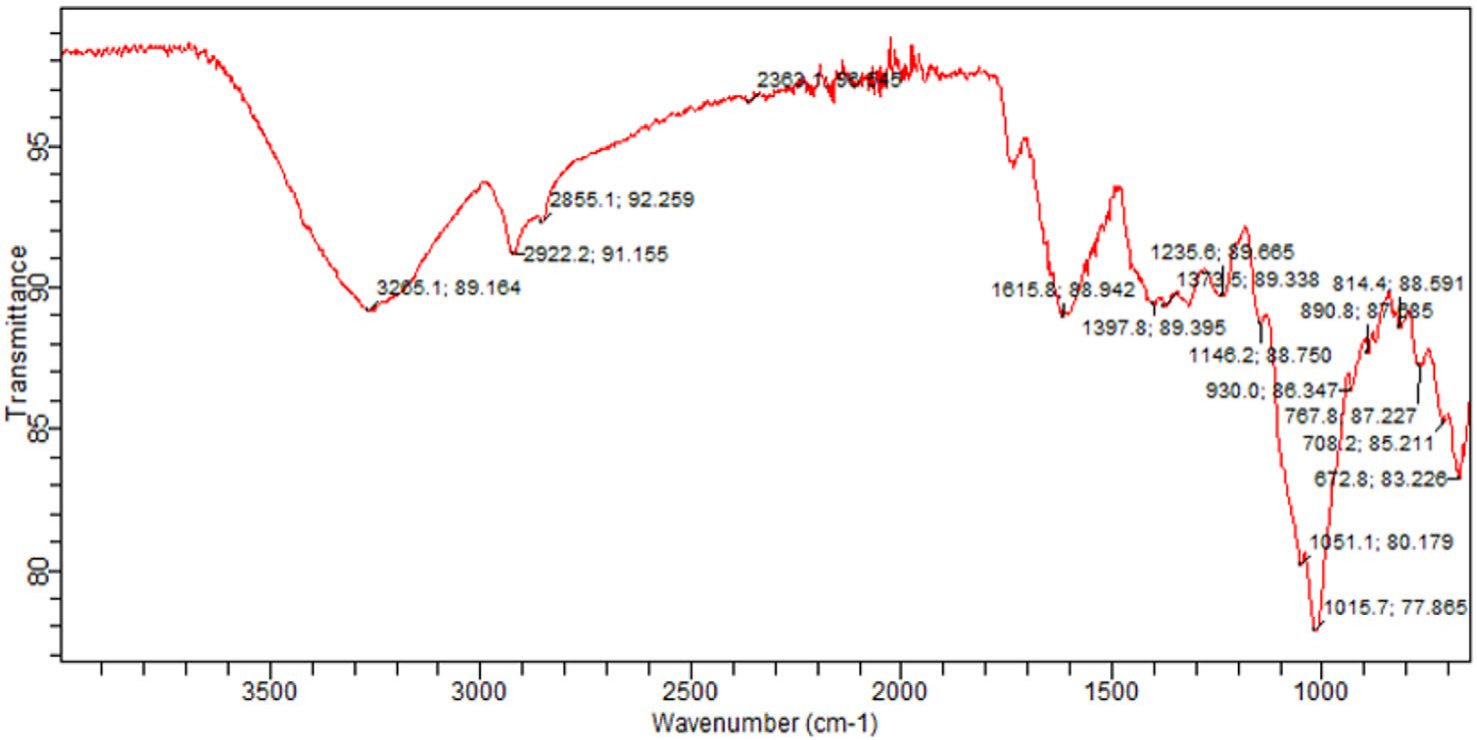
FTIR of tomato peel and pomace.

The FTIR analysis of tomato peel extract is depicted in (Figures 2, 3, 4). Notably, the characteristic peaks observed at 1604 cm^−1^ and 1541 cm^−1^ are attributed to the stretching vibrations of —C=O and C—N bonds, respectively. Additionally, peaks at 3278.2 cm^−1^ (corresponding to N—H stretching vibrations) and 2918.5 cm^−1^ (associated with C—H stretching vibrations of aliphatic groups) indicate the potential for the extract to act as both proton donors and receptors for hydrogen bonding.

In the case of FTIR analysis for tomato pomace extract, there were two weak peaks at 2924 cm^−1^, suggesting C—H stretching bonds, which were slightly shifted to lower wave numbers. The frequencies at 1636 and 1244 cm^−1^ are indicative of C=O and C—O stretching frequencies, respectively, allowing for the identification of carboxylic acids present in the tomato pomace extract.

Our results are in line with the finding of (Horuz and Belibağlı 2018), they observed C—H stretching vibrations of tomato peel extract at 2956 cm^−1^, whereas stretching vibrations of —C=O and C—N bonds at 1642 cm^−1^ and 1540 cm^−1^, respectively. Our results are also compatible with (Vorobyova et al. 2022), who observed C=O and C—O stretching of tomato pomace extract at 1670 and 1210 cm^−1^, respectively (Horuz and Belibağlı 2018) (Vorobyova et al. 2022). Similar results were also reported by (Sengar et al. 2020).

### 6.5 Physicochemical Analysis

#### 6.5.1 Texture

Texture refers to the sensory characteristics of food that can be perceived through touch, taste, and chewing. The results revealed a significant impact of the treatments on texture. It was concluded that the mean value of texture was observed in T_0_ (33.92 ± 2.86 N), the mean value of texture of jam formulations developed with tomato peel was T_3_ (27.21 ± 2.48 N), followed by T_1_ (26.12 ± 3.26 N), and T_2_ (23.70 ± 2.37 N) while the mean value of texture of jam formulations developed with tomato pomace was T_5_ (24.95 ± 2.59 N), followed by T_6_ (24.09 ± 2.53 N), and T_4_ (23.53 ± 1.78 N). The jam formulation developed with mixed tomato peel and pomace were T_7_ (19.99 ± 1.97 N), T_8_ (19.42 ± 1.21 N), and T_9_ (19.60 ± 1.87 N), (Table 8). This indicated that different treatments of jam had a significant effect on texture, and it is also influenced by the quantity of the sample.

These findings align with the results reported by (Belović et al. 2017), who investigated the textural properties of jam and calculated a value of 35.39 ± 2.81 N. They also observed the impact of sucrose on texture of jam and reported values of 20.16 ± 1.14 N (Belović et al. 2017).

#### 6.5.2 pH

The results regarding pH exhibited significant variations among the treatments presented in Table 8, namely T0, T1, T2, T3, T4, T5, T6, T7, T8, and T9. The controlled jam (T0) had the highest pH value at 4.07 ± 0.28, followed by T6 at 4.02 ± 0.39, T3 at 3.93 ± 0.37, T7 at 3.87 ± 0.36, T5 at 3.72 ± 0.35, T9 at 3.51 ± 0.33, T8 at 3.43 ± 0.31, T1 at 3.42 ± 0.31, T2 at 3.32 ± 0.30, and T4 at 3.05 ± 0.28 (as shown in Table 7).

**TABLE 7:** Texture and pH of jam samples.

Treatments (*F* value = 3.74**)	Texture				pH
	Stickiness	Work of adhesion	Firmness	Mean	
$T_{0}$	−12.25 ± 1.25	−0.015 ± 0.001	46.19 ± 1.44	33.92 ± 2.86	4.07 ± 0.28
$T_{1}$	−7.14 ± 0.73	−0.007 ± 0.001	33.27 ± 3.27	26.12 ± 3.26	3.42 ± 0.31
$T_{2}$	−7.58 ± 0.71	−0.008 ± 0.001	31.29 ± 2.87	23.70 ± 2.37	3.32 ± 0.30
$T_{3}$	−8.34 ± 0.68	−0.009 ± 0.001	32.56 ± 2.48	27.21 ± 2.48	3.93 ± 0.37
$T_{4}$	−18.42 ± 0.77	−0.020 ± 0.003	41.97 ± 0.78	23.53 ± 1.78	3.05 ± 0.28
$T_{5}$	−17.26 ± 0.79	−0.019 ± 0.004	42.23 ± 2.59	24.95 ± 2.59	3.72 ± 0.35
$T_{6}$	−17.16 ± 0.80	−0.018 ± 0.004	41.27 ± 2.53	24.09 ± 2.53	4.02 ± 0.39
$T_{7}$	−15.50 ± 0.82	−0.015 ± 0.005	35.50 ± 1.97	19.99 ± 1.97	3.87 ± 0.36
$T_{8}$	−16.15 ± 0.78	−0.016 ± 0.004	35.59 ± 3.21	19.42 ± 1.21	3.43 ± 0.31
$T_{9}$	−16.67 ± 0.75	−0.017 ± 0.004	36.29 ± 2.87	19.60 ± 1.87	3.51 ± 0.33
Mean	**−11.93 ± 0.80b**	**−0.014 ± 0.001d**	**37.91 ± 2.24a**		**3.55 ± 0.34c**

*Note:* Values are presented as mean ± SD (*n* = 3). T_0_: Control, T_1_: Jam with Tomato peel powder (15%), T_2_: Jam with Tomato peel powder (17%), T_3_: Jam with Tomato peel powder (19%), T_4_: Jam with Tomato pomace powder (15%), T_5_: Jam with Tomato pomace powder (17%), T_6_: Jam with Tomato pomace powder (19%), T_7_: Jam with Tomato peel & powder (7.5 + 7.5%), T_8_: Jam with Tomato peel & powder (8.5 + 8.5%), T_9_: Jam with Tomato peel & powder (9.5 + 9.5%). Bold values highlight the highest or most significant mean values in each comparison.

* Indicates significance at *p* ≤ 0.05.

** Indicates significance at *p* ≤ 0.01.

The findings of this study align with the observations made by Parafati et al. (2020), who concluded that the pH of the product decreases with an increase in the concentration of tomato peel. They reported a pH of 3.55 ± 0.02 for the control sample and 4.11 ± 0.01 for the treated sample with peel powder. Similarly, previous research conducted by Thilagavathi and Deepa (2011) showed comparable pH values of 3.50 ± 0.22 for standard cocoa jam and 4.17 ± 0.02 for developed cocoa jam. Additionally, Silva and colleagues (2015) found a pH value of 4.69 ± 0.02 for jam (Pa Silva et al. 2019).

#### 6.5.3 Color

The level of consumer acceptance for a food product can be determined by various important factors, including its color. Color is considered a desirable attribute for consumers when evaluating a product. The color of a food product is typically assessed using the L*, a*, and b* values in the CIELAB color system. In this system, L* represents brightness, a* represents the range from greenish to reddish tones, and b* represents the range from bluish‐to‐yellowish tones. The analysis of the jam formulations color demonstrated that the treatments had a significant impact on all aspects of the product's color. The L* values (indicating lightness) for the different jam samples, T0, T1, T2, T3, T4, T5, T6 T7, T8, and T9 were 67.25 ± 3.01, 13.97 ± 0.95, 12.42 ± 0.87, 14.93 ± 1.02, 17.34 ± 1.21, 19.15 ± 1.45, 18.09 ± 1.34, 15.76 ± 1.10, 17.98 ± 1.23, and 16.45 ± 1.19, respectively. The a* value (representing the greenish to reddish tonality) of T0 was 29.26 ± 1.32, followed by 19.14 ± 1.89, 18.88 ± 1.81, 19.75 ± 1.87, 26.43 ± 1.20, 24.84 ± 2.02, 25.67 ± 2.11, 21.23 ± 0.92, 22.78 ± 0.98, and 24.23 ± 1.08 in T1, T2, T3, T4, T5, T6 T7, T8, and T9, respectively. The b* values (representing the bluish‐to‐yellowish color) were 17.05 ± 1.71, 7.38 ± 0.71, 7.86 ± 0.76, 8.59 ± 0.82, 11.17 ± 0.98, 12.89 ± 1.03, 13.56 ± 1.12, 9.78 ± 0.92, 10.29 ± 0.98, and 11.24 ± 0.99 for T0, T1, T2, T3, T4, T5, T6, T7, T8, and T9, respectively. There was an increasing trend observed in the controlled sample and a decreasing trend in the treated samples, as shown in Table 8. Previously, Silva and colleagues (2015) reported L* values of 25.97 ± 0.20, a* values of 3.42 ± 0.07, and b* values of 3.21 ± 0.09 for jam (Pa Silva et al. 2019).

**TABLE 8:** Color of jam made of tomato peel and pomace.

Treatments (*F* value =3.36**)	Storage period (*F* value = 8.12*)					
	0 Day	7 Day	14 Day	21 Day	28 Day	Mean
$T_{0}$	8.15 ± 0.73	8.11 ± 0.72	8.03 ± 0.71	7.07 ± 0.72	7.05 ± 0.61	**7.67 ± 0.68a**
$T_{1}$	8.45 ± 0.76	8.26 ± 0.75	7.23 ± 0.67	7.14 ± 0.66	7.09 ± 0.62	**7.63 ± 0.65a**
$T_{2}$	7.35 ± 0.67	7.41 ± 0.68	7.16 ± 0.66	7.08 ± 0.61	7.01 ± 0.61	**7.19 ± 0.64b**
$T_{3}$	7.15 ± 0.66	7.46 ± 0.68	7.03 ± 0.61	6.81 ± 0.58	6.25 ± 0.40	**6.93 ± 0.65d**
$T_{4}$	7.35 ± 0.62	7.13 ± 0.49	7.06 ± 0.42	6.75 ± 0.41	6.23 ± 0.37	**6.89 ± 0.44e**
$T_{5}$	7.19 ± 0.48	7.05 ± 0.45	6.56 ± 0.42	6.43 ± 0.39	6.22 ± 0.36	**6.68 ± 0.40f**
$T_{6}$	7.23 ± 0.58	7.25 ± 0.55	7.12 ± 0.48	6.85 ± 0.45	6.79 ± 0.42	**7.04 ± 0.43c**
$T_{7}$	7.15 ± 0.47	7.16 ± 0.50	7.02 ± 0.40	6.81 ± 0.42	6.66 ± 0.43	**6.96 ± 0.45d**
$T_{8}$	7.03 ± 0.72	7.11 ± 0.65	6.56 ± 0.43	6.25 ± 40	6.21 ± 0.31	**6.63 ± 0.46f**
$T_{9}$	7.05 ± 0.60	7.16 ± 0.64	7.01 ± 0.60	6.66 ± 0.43	6.26 ± 0.38	**6.82 ± 0.46e**
Mean	**7.41 ± 0.67a**	**7.41 ± 0.67a**	**6.37 ± 0.58d**	**6.78 ± 0.62b**	**6.57 ± 0.61c**	

*Note:* Values are presented as mean ± SD (*n* = 3). Values imparted by different letters in columns and rows were significantly different. (*p* ≤ 0.05). Bold values highlight the highest or most significant mean values in each comparison.

* Indicates significance at *p* ≤ 0.05.

** Indicates significance at *p* ≤ 0.01.

### 6.6 Sensory Evaluation of Low‐Caloric Jam Made of Tomato Peel & Pomace

For a product's acceptance and marketability, hedonic response is predictable. A positive sensory response confirms consumer approval and faith in the manufactured product. Various sensory qualities, such as color, flavor, sweetness, sourness, and general acceptability, were assessed for the low‐caloric jam.

The first perception of consumer to receive any food product referred as appearance and showed momentous by treatment. However, there interactive effect imparted non‐significant effect. Means of the appearance acceptability (Table 9) showed significant deference between 8.06 ± 0.73 to 6.93 ± 0.65 in T0 to T_9_. Similarly, a significant decline was observed from 7.59 ± 0.69 to 7.02 ± 0.62 at 0th to 28th days, correspondingly.

**TABLE 9:** Flavor of jam made of tomato peel and pomace.

Treatments (*F* value = 4.35**)	Storage period (*F* value = 8.49*)					
	0 Day	7 Day	14 Day	21 Day	28 Day	Mean
$T_{0}$	8.45 ± 0.76	8.26 ± 0.75	7.23 ± 0.67	7.14 ± 0.66	7.09 ± 0.62	**7.63 ± 0.65a**
$T_{1}$	7.26 ± 0.65	7.18 ± 0.66	6.26 ± 0.40	6.15 ± 0.23	6.05 ± 0.21	**6.58 ± 0.43e**
$T_{2}$	8.15 ± 0.73	7.76 ± 0.69	7.25 ± 0.67	7.13 ± 0.73	7.09 ± 0.72	**7.47 ± 0.74b**
$T_{3}$	7.03 ± 0.72	7.11 ± 0.65	6.56 ± 0.43	6.25 ± 40	6.21 ± 0331	**6.63 ± 0.46e**
$T_{4}$	7.25 ± 0.67	7.49 ± 0.74	7.12 ± 0.60	6.27 ± 0.41	6.12 ± 0.30	**6.84 ± 0.48d**
$T_{5}$	7.26 ± 0.65	7.18 ± 0.66	6.26 ± 0.40	6.15 ± 0.23	6.05 ± 0.21	**6.58 ± 0.43e**
$T_{6}$	7.24 ± 0.65	7.11 ± 0.6	6.89 ± 0.60	6.56 ± 0.54	6.39 ± 0.49	**6.83 ± 0.58d**
$T_{7}$	7.19 ± 0.61	7.45 ± 0.64	6.84 ± 0.58	6.47 ± 0.52	6.21 ± 0.42	**6.82 ± 0.57d**
$T_{8}$	7.65 ± 0.70	7.27 ± 0.66	7.36 ± 0.64	6.59 ± 0.57	6.37 ± 0.46	**7.04 ± 0.61c**
$T_{9}$	7.25 ± 0.66	7.14 ± 0.61	6.34 ± 0.45	6.23 ± 0.44	6.01 ± 0.40	**6.61 ± 0.60e**
Mean	**7.47 ± 0.24a**	**7.39 ± 0.67b**	**6.81 ± 0.56c**	**6.49 ± 0.48d**	**6.35 ± 0.52e**	

*Note:* Values are presented as mean ± SD (*n* = 3). Values imparted by different letters in columns and rows were significantly different. (*p* ≤ 0.05). Bold values highlight the highest or most significant mean values in each comparison.

* Indicates significance at *p* ≤ 0.05.

** Indicates significance at *p* ≤ 0.01.

The first sensory character for the selection of a product received by customer is a color of the product. The acceptability of color (Table 10) imparted non‐momentous effects during storage 0th to 28th days from 7.41 ± 0.67 to 6.57 ± 0.61. Likewise, treatments revealed significant differences in T_0_ and T_9_, respectively.

**TABLE 10:** Aroma of jam made of tomato peel and pomace.

Treatments *F* value (3.56**)	Storage period (*F* value = 7.36*)					
	0 Day	7 Day	14 Day	21 Day	28 Day	Mean
$T_{0}$	6.25 ± 0.40	6.17 ± 0.34	6.05 ± 0.30	6.10 ± 0.31	6.02 ± 0.30	**6.13 ± 0.31c**
$T_{1}$	6.75 ± 0.45	6.85 ± 0.47	6.25 ± 0.38	6.14 ± 0.34	6.08 ± 0.30	**6.41 ± 0.42a**
$T_{2}$	6.48 ± 0.42	6.33 ± 0.38	6.31 ± 0.39	6.19 ± 0.35	6.13 ± 0.31	**6.28 ± 0.28b**
$T_{3}$	6.23 ± 0.37	6.12 ± 0.35	6.06 ± 0.30	6.01 ± 0.29	5.78 ± 0.22	**6.04 ± 0.30d**
$T_{4}$	6.23 ± 0.35	6.13 ± 0.32	6.15 ± 0.33	6.07 ± 0.31	6.03 ± 0.31	**6.11 ± 0.32c**
$T_{5}$	6.75 ± 0.55	6.46 ± 0.44	6.25 ± 0.43	6.20 ± 0.32	6.14 ± 0.32	**6.35 ± 0.46a**
$T_{6}$	6.45 ± 0.40	6.78 ± 0.56	6.22 ± 0.32	6.16 ± 0.28	6.09 ± 0.31	**6.34 ± 0.45a**
$T_{7}$	6.41 ± 0.58	6.26 ± 0.61	6.24 ± 0.64	5.96 ± 0.58	5.56 ± 0.41	**6.08 ± 0.3d**
$T_{8}$	6.31 ± 0.54	6.15 ± 0.46	6.04 ± 0.63	5.87 ± 0.55	5.66 ± 0.36	**6.06 ± 0.15d**
$T_{9}$	6.51 ± 0.30	6.13 ± 0.36	5.96 ± 0.34	5.78 ± 0.45	5.56 ± 0.21	**6.04 ± 0.31d**
Mean	**6.43 ± 0.42a**	**6.33 ± 0.41b**	**6.15 ± 0.40c**	**6.20 ± 0.54c**	**5.90 ± 0.45d**	

*Note:* Values are presented as mean ± SD. (*n* = 3). Values imparted by different letters in columns and rows were significantly different. (*p* ≤ 0.05). Bold values highlight the highest or most significant mean values in each comparison.

* Indicates significance at *p* ≤ 0.05.

** Indicates significance at *p* ≤ 0.01.

The flavor is the result of the identification of taste through the taste bud present in the oral cavity, and epithelial tissues are responsible for the perception of aromatic compounds in the olfactory present in the mouth. The treatment score for flavor acceptability revealed in Table 9 was imparted non‐momentous differences in T_0_ (7.63 ± 0.65) followed by 7.47 ± 0.74, 6.84 ± 0.48, 6.83 ± 0.58, 6.82 ± 0.57, 7.04 ± 0.61, 6.63 ± 0.46, 6.61 ± 0.60, 6.58 ± 0.43, and 6.58 ± 0.43 in T_2_, T_8_, T_4_, T_6_, T_7_, T_3_, T_9_, T_1_, and T_5_, respectively. Whereas, the mean score of flavor during storage was affected significantly between 7.47 ± 0.24 and 6.35 ± 0.52 at the beginning (0th) and at the termination (28th) days, respectively.

The maximum score of aroma acceptability was assigned to (T_1_) on 6.41 ± 0.42, trailed by T_5_ (6.35 ± 0.46), T_6_ (6.34 ± 0.45), T_0_ (6.28 ± 0.28), T_0_ (6.13 ± 0.31), T_4_ (6.11 ± 0.32), T_7_ (6.08 ± 0.3), T_8_ (6.06 ± 0.15), T_9_ (6.04 ± 0.31), and T_3_ (6.04 ± 0.30). The score of aroma was also affected during storage, decreasing from 6.36 ± 0.33 to 5.94 ± 0.24 from the 0th to 28th days, as shown in Tables 10, 11.

**TABLE 11:** Overall acceptability of jam made of tomato peel and pomace.

Treatments *F* value (3.78**)	Storage period (*F* value = 7.23*)					
	0 Day	7 Day	14 Day	21 Day	28 Day	Mean
$T_{0}$	7.35 ± 0.67	7.41 ± 0.68	7.16 ± 0.66	7.08 ± 0.61	7.01 ± 0.61	**7.19 ± 0.64**
$T_{1}$	7.15 ± 0.66	7.46 ± 0.68	7.03 ± 0.61	6.81 ± 0.58	6.25 ± 0.40	**6.93 ± 0.65**
$T_{2}$	7.19 ± 0.61	7.45 ± 0.64	6.84 ± 0.58	6.47 ± 0.52	6.21 ± 0.42	**6.82 ± 0.57**
$T_{3}$	7.65 ± 0.70	7.27 ± 0.66	7.36 ± 0.64	6.59 ± 0.57	6.37 ± 0.46	**7.04 ± 0.61**
$T_{4}$	7.25 ± 0.66	7.14 ± 0.61	6.34 ± 0.45	6.23 ± 0.44	6.01 ± 0.40	**6.61 ± 0.60**
$T_{5}$	7.24 ± 0.65	7.11 ± 0.6	6.89 ± 0.60	6.56 ± 0.54	6.39 ± 0.49	**6.83 ± 0.58**
$T_{6}$	7.25 ± 0.67	7.15 ± 0.63	7.19 ± 0.65	7.13 ± 0.64	7.09 ± 0.60	**7.15 ± 0.64**
$T_{7}$	7.05 ± 0.60	7.16 ± 0.64	7.01 ± 0.60	6.66 ± 0.43	6.26 ± 0.38	**6.82 ± 0.46**
$T_{8}$	6.25 ± 0.38	6.56 ± 0.44	6.23 ± 0.37	6.12 ± 0.33	6.06 ± 0.29	**6.27 ± 0.37**
$T_{9}$	7.24 ± 0.66	6.59 ± 0.45	6.46 ± 0.42	6.24 ± 0.36	6.18 ± 0.34	**6.54 ± 0.46**
Mean	**7.16 ± 0.64a**	**7.13 ± 0.61a**	**6.84 ± 0.56b**	**6.58 ± 0.47c**	**5.78 ± 0.32d**	

*Note:* Values are presented as mean ± SD (*n* = 3). Values imparted by different letters in columns and rows were significantly different. (*p* ≤ 0.05). Bold values highlight the highest or most significant mean values in each comparison.

* Indicates significance at *p* ≤ 0.05.

** Indicates significance at *p* ≤ 0.01.

The depicted result in Table 11 showed the influence of various treatments on jam on overall acceptability was significant among various treatments and different storage intervals. Means of the overall acceptability showed significant deference between 7.19 ± 0.64 and 6.54 ± 0.46 in T_0_ to T_9_, respectively. However, the mean score of overall acceptability during storage was affected significantly between 7.16 ± 0.64 and 5.78 ± 0.32 at the beginning (0th) and at the termination (28th) days, respectively.

The results clearly show that the tomato peel and pomace powder did not have any negative effects on the product. The sensory evaluation of current research is in line with the findings of Belović et al. (2017); they reported that with the addition of substitutes, the sensory properties of the product increased. The values ranged from 8.0 to 5.3. The observations were similar to the findings of Patel and Naik (2013) in pineapple and banana jam, Priya et al. (2010) in mixed fruit jam, and Relekar et al. (2010) in sapota jam (Patel and Naik 2013).

## 7 Strengths and Limitations

Researchers advocate for the consumption of tomato peel and seed waste, a move that supports the global initiative of minimizing food waste and environmental footprint. Through the development of a low‐calorie jam, the study meets increased consumer demand for healthier alternatives to the standard high‐sugar fruit preserves. Tomato waste is a rich source of bioactive compounds like lycopene, dietary fiber, and phenolics, and their incorporation could increase the nutrient and functional value of the jam. Use of agro‐industrial by‐products can reduce costs of production, resulting in economic benefits to small‐ and medium‐scale food processors. The research probably tests a set of parameters including physicochemical, sensorial, and nutrient properties, offering a holistic picture of product behavior. If the study lacks large‐scale or diverse consumer testing, the generalizability of sensory acceptance data may be limited. Storage stability or shelf life of the low‐calorie jam may not be thoroughly studied over a long time, which is crucial for commercial purposes. Tomato waste composition varies with cultivar, processing procedure, and season, and thus may influence the reproducibility and uniformity of the product. It can concentrate on laboratory‐scale or pilot‐scale formulation, and subsequent studies to validate scalability to commercial scale are required. Application of tomato waste to food products has the potential to pose regulatory and labeling issues in some areas, which were not discussed in detail.

## 8 Future Recommendations

Investigation is also needed to streamline pre‐treatment and extraction of tomato waste to achieve the highest retention of bioactive compounds and best texture and flavor of the end product, jam. Long‐term stability experiments under multiple conditions of storage must be done to monitor physicochemical, microbiological, and sensorial changes over time. Sensory evaluations with expanded consumer panels of varied age, gender, and cultural groups are needed to evaluate market acceptability across a greater range. Production trial work on pilot and industrial scales must be done to test the feasibility, uniformity, and cost value of the formulation at commercial scales. Future research may investigate the application of natural antimicrobials or antioxidants (such as plant extracts and essential oils) to maintain product shelf life and safety without artificial additives. In vivo or clinical studies may also be done to prove the benefits of tomato‐waste‐enriched jam to human health, specifically regarding antioxidant properties, glucose response, or gut health. Life cycle analysis (LCA) or carbon footprint analysis of the product may yield quantifiable information on its environmental advantages and substantiate claims of sustainability. Merging tomato waste with other fruit by‐products has the potential to complement the nutrient content, minimize waste, and provide unique flavor profiles to diversify products.

## 9 Conclusions

In conclusion, food waste valorization has gained considerable attention due to its potential health benefits, and tomato, known for its bioactive constituents, was used in this study to develop low‐calorie jam from tomato peel and pomace. The analysis of tomato peel and pomace revealed varying moisture, ash, fat, fiber, protein, and NFE content. Both tomato peel and pomace exhibited good antioxidant activity, with the peel demonstrating better performance. The study found that different solvents and time intervals influenced the extraction of various bioactive compounds, with methanol generally yielding the highest values for most parameters. Among the developed jam formulations, texture, pH, acid value, and color properties varied significantly. However, there were no significant differences in the hedonic response, except for color, and the color scores of the jam decreased during storage. These findings demonstrate the potential of utilizing tomato peel and pomace in the development of functional food products, contributing to the reduction of food waste and promoting a sustainable and health‐conscious approach.

## Author Contributions

**Muhammad Hammad:** writing – original draft (equal). **Amar Shankar:** visualization (equal). **G. V. Sivaprasad:** formal analysis (equal). **Qaswaa Yousif Jameel:** validation (equal). **Faiyaz Ahmed:** data curation (equal), software (equal). **Ali Imran:** supervision. **Muhammad Afzaal:** methodology. **Amar Shankar:** data curation. **Musarrat Rasheed:** review editing. **Usman Naeem:** software. **Mohd Asif Shah:** formal analysis.

## Consent

All authors are willing for publication of this manuscript.

## Conflicts of Interest

The authors declare no conflicts of interest.

## Data Availability

The datasets generated and/or analyzed during the current study are available from the corresponding author upon reasonable request.
